# Incidental Detection of Unilateral Mandibular Bone Thinning Without Clinical Asymmetry in an Adult Female: A Rare Radiographic Finding

**DOI:** 10.7759/cureus.99082

**Published:** 2025-12-12

**Authors:** Shubham K Srivastava, Chinmoy Sikdar, Akshim Rana, Shitij Srivastava, Samsul A Choudhury

**Affiliations:** 1 Department of Prosthodontics, Sardar Patel Post Graduate Institute of Dental and Medical Sciences, Lucknow, IND; 2 Conservative Dentistry and Endodontics, Career Post Graduate Institute of Dental Sciences and Hospital, Lucknow, IND

**Keywords:** diagnostic imaging, mandibular hypoplasia, mandibular morphology, orthopantomograph, prosthodontic planning

## Abstract

Unilateral mandibular thinning is an uncommon radiographic finding, particularly when unaccompanied by facial asymmetry or functional disturbances. This observational report describes the incidental detection of marked unilateral thinning of the mandibular body and ramus in a 40-year-old asymptomatic female during routine panoramic radiography, with no immediate intervention required. Clinical evaluation revealed no facial asymmetry, occlusal discrepancy, or history of trauma, surgery, infection, or systemic disease. Radiographic features suggested a long-standing, non-progressive skeletal variation most consistent with a developmental anomaly, without evidence of cortical breach, expansile remodeling, or destructive pathology. Although cone-beam computed tomography was considered for more detailed assessment, it was deemed unnecessary given the absence of clinical or radiographic indicators of pathology. Recognition of such incidental findings is essential to prevent misdiagnosis and to guide clinical and surgical planning, including implant placement considerations. Periodic radiographic follow-up was recommended to ensure stability of the mandibular architecture.

## Introduction

Radiographic evaluation plays a central role in dental diagnostics and often uncovers clinically silent anomalies that might otherwise remain unrecognized. The mandible, as the largest and most structurally robust facial bone, typically exhibits a high degree of bilateral symmetry, although deviations from this symmetry have been documented in developmental and condylar anomalies [1]. Such variations are uncommon and warrant careful assessment, particularly when they involve alterations in cortical or medullary bone morphology. Unilateral thinning of the mandibular body or ramus is an especially rare finding, particularly in the absence of facial asymmetry, occlusal disturbance, or temporomandibular dysfunction, in contrast to more evident conditions such as condylar aplasia or hypoplasia [2].

Most mandibular asymmetries arise from identifiable causes, including congenital or developmental disturbances, metabolic disorders, trauma, infection, or neoplastic processes, with condylar anomalies representing a significant proportion of these variations [3]. In contrast, idiopathic or developmental unilateral mandibular thinning without associated clinical signs is infrequently reported, and the existing literature primarily addresses condylar deviations or dysplasia rather than uniform cortical thinning [4]. The present case appears to represent a non-progressive developmental variation, with no history or radiographic evidence suggestive of minor remodeling or acquired bone loss, contributing to the limited documentation of uniform unilateral cortical thinning without condylar involvement.

The incidental detection of such an anomaly during routine panoramic imaging highlights the broader diagnostic capacity of conventional radiography, which is well validated for identifying mandibular asymmetry and dimensional discrepancies [5]. While panoramic radiography is an effective first-line screening tool due to its accessibility, low radiation dose, and ability to capture the entire mandible in a single projection, it can underestimate subtle cortical thinning or minor trabecular variations because of its two-dimensional nature and superimposition of structures. Cone-beam computed tomography (CBCT) provides superior three-dimensional visualization and higher spatial resolution, enabling more precise assessment of cortical thickness and differentiation between developmental variation and pathological bone loss [6,7]. However, in asymptomatic patients without clinical indicators of pathology, CBCT may be reserved for cases where panoramic imaging is inconclusive or where surgical planning necessitates detailed cortical mapping.

Recognition of these uncommon skeletal variations is clinically relevant, as they may influence diagnostic interpretation, surgical planning, and long-term treatment outcomes. This report presents an incidental case of unilateral mandibular bone thinning in an asymptomatic adult female, contributing to the sparse body of evidence on subtle radiographic presentations of mandibular asymmetry.

## Case presentation

A 40-year-old female patient presented for routine dental evaluation. Her medical, surgical, and family histories were noncontributory, and she denied any history of facial trauma, jaw surgery, systemic illness, or temporomandibular complaints. Extraoral inspection revealed a symmetrical facial profile without swelling, deviation, or soft-tissue abnormality. Intraoral examination demonstrated a normal occlusal scheme, with no mucosal lesions or functional disturbances.

**Figure 1:**
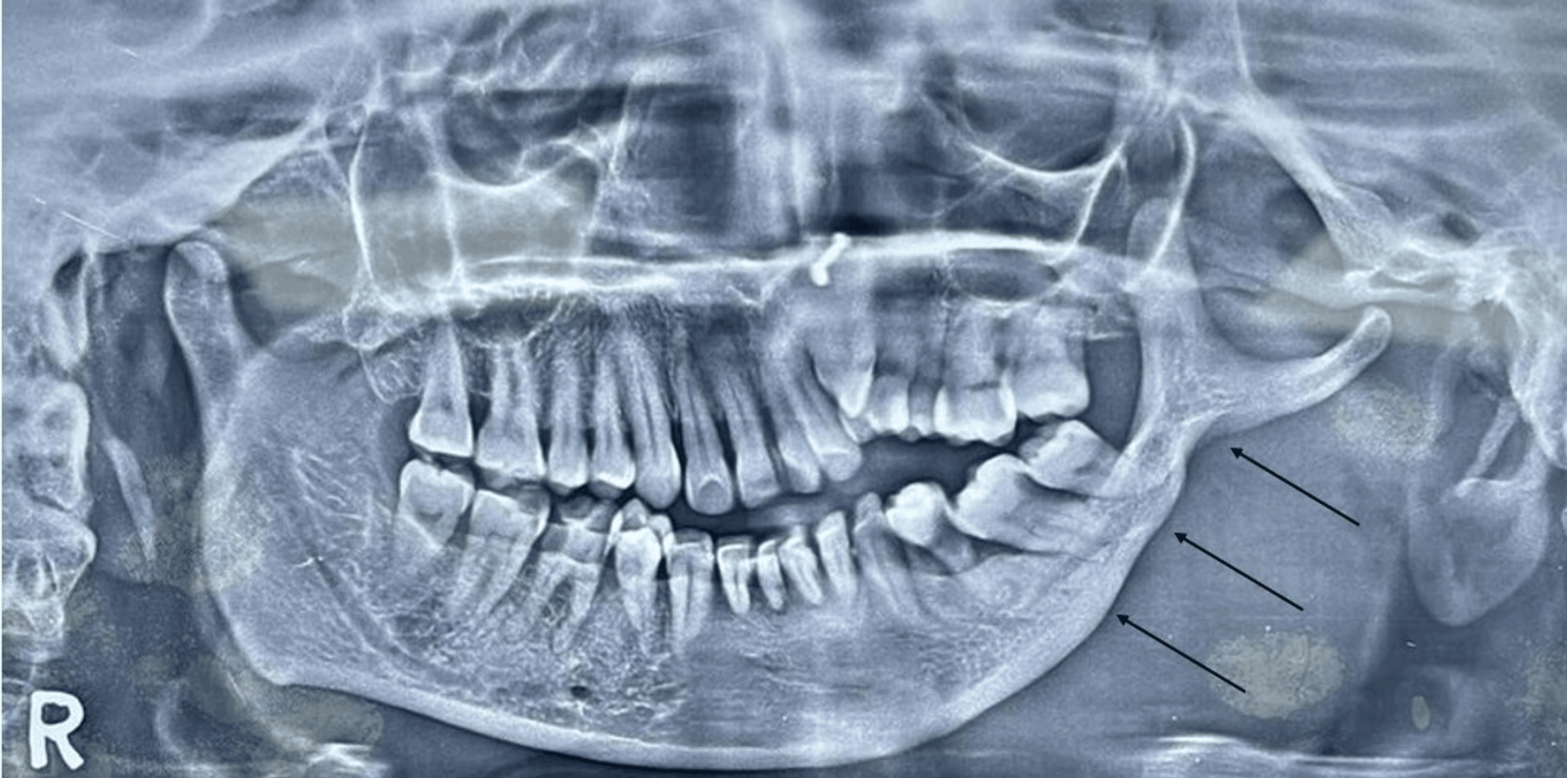
Unilateral thinning of the left mandibular body Panoramic radiograph showing unilateral thinning of the left mandibular body and angle (third quadrant) (arrows). Cortical integrity is preserved, and there is no associated radiolucency, expansion, or evidence of active pathology. The finding was incidental and lacked clinical correlates of asymmetry or dysfunction, suggesting a long-standing, likely developmental skeletal variation.

A panoramic radiograph obtained during routine screening revealed marked unilateral thinning of the left mandibular body (third quadrant), extending posteriorly toward the angle and partially involving the ramus. The inferior cortical margin was continuous and well defined, with no evidence of cortical breach, erosion, periosteal reaction, or focal destructive change. Compared with the contralateral side, the trabecular bone in the affected region demonstrated reduced density and apparent volume; however, there were no radiolucent, radiopaque, or mixed lesions, and the mandibular canal remained traceable and preserved. Condylar morphology and temporomandibular joint spaces were within normal limits bilaterally (Figure 1).

Cortical thickness was measured on the panoramic radiograph using calibration-adjusted digital linear tools within the imaging software, with a scale of 0.1 mm per pixel to ensure reproducibility. Measurements were taken at standardized anatomical landmarks (mid-body and mandibular angle) perpendicular to the cortical margins. Each measurement was repeated three times, and the average values were compared with published reference norms for adult mandibular cortical thickness to improve reliability [8].

Although panoramic radiography provided adequate initial screening, CBCT is considered the gold standard for detailed three-dimensional assessment when cortical defects, dehiscence, or fenestration are suspected; it offers higher spatial resolution for subtle cortical and trabecular evaluation [9]. In this asymptomatic patient, CBCT was deemed unnecessary, as panoramic findings showed clear cortical margins, uniform trabeculation, and no evidence of progressive change.

In the absence of clinical symptoms, progressive radiographic features, or indicators of a pathologic process, the radiographic presentation was interpreted as a benign, non-progressive developmental variant. The patient was informed and reassured, and no immediate intervention was indicated. Periodic radiographic follow-up was recommended to document stability.

## Discussion

Unilateral cortical and trabecular reduction of the mandibular body or ramus is an uncommon radiographic finding, usually associated with identifiable developmental, traumatic, infectious, metabolic, or neoplastic causes. Most reported cases of mandibular asymmetry present with clinical correlates such as facial deviation or occlusal discrepancies, particularly in conditions like condylar aplasia or developmental dysmorphology [10]. The present case is unusual because it demonstrates significant cortical and trabecular thinning without any associated clinical asymmetry, placing it outside the typical spectrum of described mandibular deformities [11].

Developmental variations of mandibular morphology have been attributed to asymmetrical basal bone growth, condylar growth disturbances, or congenital skeletal differences, with several reports highlighting condylar aplasia or hypoplasia as primary contributors to mandibular imbalance [12]. In this case, preserved cortical continuity, absence of focal radiolucency or sclerosis, normal condylar morphology, and lack of periosteal reaction support a non-progressive developmental variant rather than an acquired pathology. The uniform reduction in bone thickness further argues against cystic lesions, fibrous dysplasia, chronic osteomyelitis, or benign/malignant neoplasms, which typically present with radiographic expansion or destructive changes [13].

Although trauma-induced remodeling can occasionally mimic cortical reduction, the absence of trauma history and radiographic signs of callus formation make this unlikely. Metabolic bone disorders were also considered but deemed improbable given the unilateral nature of the finding and the absence of systemic manifestations. Idiopathic mandibular resorption generally progresses over time with notable morphological deterioration, unlike the stable appearance observed in this patient [14].

This anomaly underscores the diagnostic value of panoramic radiography, which remains a widely used screening tool with strong reproducibility for assessing mandibular asymmetry and dimensional relationships [15]. CBCT provides superior three-dimensional visualization and detailed assessment of cortical and trabecular morphology; however, its use is typically reserved for cases where pathology is suspected or for surgical planning. In asymptomatic patients with stable cortical boundaries, as in this case, additional 3D imaging may not be required [16]. Limitations of the present report include the absence of volumetric imaging and quantitative metrics such as precise cortical thickness and trabecular density, which restricts objective assessment and longitudinal evaluation.

Recognition of such anatomical variations is clinically important for oral surgeons, implantologists, and prosthodontists. Reduced mandibular thickness can influence treatment planning, including implant placement, osteotomy approaches, and the low but present risk of pathological fracture. Awareness of benign developmental cortical reduction helps prevent misdiagnosis and avoids unnecessary interventions. This case contributes to the limited literature describing uniform cortical thinning without clinical asymmetry and emphasizes the importance of integrating radiographic findings with clinical evaluation to differentiate normal variants from pathology.

## Conclusions

Unilateral mandibular bone thinning without associated clinical asymmetry is a rare and often incidental radiographic finding. In the present case, preserved cortical integrity, the absence of destructive features, and the lack of symptoms supported a benign, likely developmental skeletal variation rather than an acquired pathological process. This underscores the importance of thorough radiographic evaluation in routine dental imaging, as clinically silent anatomical variations may influence future diagnostic interpretation and surgical or implant-related treatment planning. Awareness of such developmental presentations enables clinicians to differentiate normal variants from disease and avoid unnecessary investigations or interventions. While a formal follow-up was not required for this asymptomatic patient, periodic monitoring would be indicated if clinical symptoms develop or radiographic changes occur, such as new pain, progressive radiolucency, or cortical breach, to ensure long-term stability.
